# Predicting mandibular growth increment on the basis of cervical vertebral dimensions in Iranian girls

**DOI:** 10.1186/2196-1042-14-3

**Published:** 2013-05-20

**Authors:** Mahkameh Moshfeghi, Hajir Rahimi, Hoda Rahimi, Mahtab Nouri, Alireza Akbarzadeh Bagheban

**Affiliations:** 1 Department of Oral and Maxillofacial Radiology, Kerman University of Medical Sciences, Kerman, , Iran; 2 Department of Orthodontics, Islamic Azad University, Dental Branch, Tehran, , Iran; 3 Department of Oral and Maxillofacial Radiology, Shahid Beheshti University of Medical Sciences, Tehran, , Iran; 4 Department of Orthodontics, Research Institute of Dental Sciences, Shahid Beheshti University of Medical Sciences, Tehran, , Iran; 5 Department of Basic Sciences, Faculty of Rehabilitation Sciences, Shahid Beheshti University of Medical Sciences, Tehran, , Iran

## Abstract

**Background:**

The purpose of this longitudinal study was to establish an equation to predict incremental mandibular length on the basis of the analysis of the cervical vertebrae on a single cephalometric radiograph and to compare the predictive accuracy with the method by Mito et al.

**Methods:**

Data consist of a group of 33 Iranian girls, 9 to 11 years old with two lateral cephalometric radiographs taken at a 24-month interval. For each individual, on the lateral cephalometric radiographs, points and lines for the description of the morphologic characteristics of the third and fourth cervical vertebral bodies were traced and measured. The real mandibular length increment (MLI) in this period was determined by the difference between the second (24 months) and first (baseline) radiographs: MLI = Ar-Pog (second) − Ar-Pog (first). An equation was determined to calculate mandibular length increments on the basis of the measurements in the third and fourth cervical vertebral bodies. The predictive accuracy was assessed using multiple regression analysis.

**Results:**

The adjusted *R*^2^ for this equation was 54.9% which is a reliable value for evaluating prediction accuracy .The average error between the predicted increment and the actual increment was 0.149 mm for our method and 5.87 mm for the method by Mito et al.

**Discussion:**

There are two items that contributed to easier and better prediction accuracy in our equation: (1) higher *R*^2^ and (2) fewer independent variables. In our subjects, the prediction accuracy was lower when using Mito et al.'s method, which could be due to genetic and environmental factors and selected age range.

**Conclusion:**

These results indicate that cervical vertebral measurements, obtained in lateral cephalograms, are able to predict properly the mandibular growth potential.

## Background

The prediction of mandibular growth potential (GP) provides valuable information for treatment planning and evaluating occlusal stability after treatment [1]. One of the main concerns which has not been of much interest during the past few decades is the issue of treatment timing. However, the maturational stage of the individual is assumed to be of great importance in contemporary diagnosis and treatment planning [2].

In the past 3 decades, the issue of the correlation between the cervical vertebral maturation (CVM) and mandibular growth has received an increasing attention. The CVM method has proved to be an effective and reliable clinical method for the assessment of mandibular skeletal maturation in growing children [3-12].

According to the results of several clinical studies, the greatest response to functional jaw orthopedics occurs during circumpubertal growth period when mandibular growth is at its peak [7-11]. Therefore, the evaluation of mandibular skeletal maturation and growth potential of each individual provides essential information for the anticipation of treatment results in this period [4].

Mito et al. [11] developed a formula to predict mandibular GP on the basis of cervical vertebral bone age in Japanese girls. In another study, Chen et al. [12] established an equation to predict incremental mandibular length on the basis of cervical vertebrae and compared the corresponding predictive accuracy with other methods. However, in all these studies, the prediction accuracy is not compared individually with the actual mandibular growth in a distinct group but rather in separate groups.

The purpose of this longitudinal study was to establish a simple method of mandibular length prediction using a regression equation on the basis of cervical vertebrae in Iranian girls and to compare the predictive accuracy with the method of Mito et al. [11].

## Methods

The sample analyzed in this longitudinal study consisted of 33 Iranian girls, 9 to 11 years old with two lateral cephalometric radiographs taken at a 24-month interval. The children were selected from the patient files of the Orthodontics Department, School of Dentistry, at the Qazvin University of Medical Sciences, Iran, based on the growth study of Qazvin City population. Written informed consent was obtained from the patient for publication of this case report and accompanying images. A copy of the written consent is available for review by the Editor-in-Chief of this journal. Ethical committee of Qazvin University of Medical Sciences approved the ethical concerns of this study. The samples were selected according to the following criteria:

(a) Angle's Class I molar and canine relationship

(b) Reasonably well-aligned arches with no vertical and horizontal dental discrepancy

(c) No systemic disease that could affect general development

(d) No history of any orthodontic treatment

(e) Pleasant soft tissue profile

(f) Cervical vertebral maturation stage (CVMS) I or CVMS II

The CVM stages were considered according to the definition by Baccetti et al. [7]. All the 66 lateral cephalograms were examined to derive the prediction equation.

### Cephalometric analysis

#### Cervical vertebral bodies

**Figure 1:**
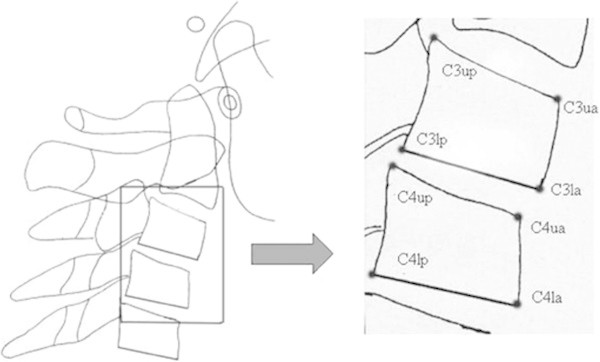
Cephalometric landmarks for the quantitative analysis of C3 and C4.

On the lateral cephalometric radiographs, the following points and lines for the description of the morphologic characteristics of the cervical vertebral bodies (Figure 1) were traced and measured using WIXWIN 2000 software (Gendex Dental System, Des Plaines, IL, USA). Computed cephalometric tracing are as accurate as manual tracing with the advantage of being less time-consuming. Furthermore, the software takes the magnification factor into account automatically.

•C3up, C3ua: the most superior points of the posterior and anterior borders of the body of C3, respectively.

•C3lp, C3la: the most posterior and the most anterior points on the lower border of the body of C3, respectively.

•C4up, C4ua: the most superior points of the posterior and anterior borders of the body of C4, respectively.

•C4lp, C4la: the most posterior and the most anterior points on the lower border of the body of C4, respectively.

•AH3, AH4 (anterior vertebral body height of the C3 and C4): the distance between C3ua and C3la and the distance between C4ua and C4la, respectively.

•PH3, PH4 (posterior vertebral body length of the C3 and C4): the distance between C3up and C3lp and the distance between C4up and C4lp, respectively.

•AP3, AP4 (anteroposterior vertebral body length of the C3 and C4): the distance between C3la and C3lp and the distance between C4la and C4lp, respectively [12].

#### Mandible

For each individual, the distance between articulare and pogonion (Ar-Pog) was assessed on both radiographs, and the mandibular length increment (MLI) in this period was determined by the difference between the second (24 months) and first (baseline) radiographs:

MLI = Ar-Pog (second) − Ar-Pog (first).

The data obtained from all 66 cephalograms were analyzed by statistical package SPSS (Chicago, IL, USA) using multiple regression analysis. In this analysis, the values of the MLI were the dependent variables, and the values of the cervical vertebrae at the first radiographs were the independent variables. The selections of the independent variables were performed using the stepwise method. Jackknife procedure was used to assess the cross-validation of our model.

To determine the measurement errors, 10 subjects were selected randomly, and cephalometric radiographs of each stage (a total 20 radiographs) were traced and measured 10 days later. The reliability of the measurements was evaluated using paired samples *t* test.

### The predictive accuracy

The adjusted *R*^2^ from the regression was used as a criterion to predict accuracy. The proposed formula by Mito et al. [11], which is suitable to predict mandibular growth potential in skeletal Class I patients, was applied on the subjects to compare the predictive accuracy with our equation. We used their definitions for measurements and calculated the error between the predicted GP and the actual growth.

## Results

### Measurements

Means, standard deviations, and the results of paired sample *t* test between the first and second radiographs, as well as mean differences between the measurements in two stages are shown in Table 1. As shown in this table, the mandible exhibited significant growth during this 24-month interval which coincided with the greatest growth in the anterior height of C3 (AH3).

**Table 1:** Mean ± SD of C3 and C4 measurements

**Variable name**	**First radiograph**	**Second radiograph**	**Mean difference**	***P *** **value**
AH3 (mm)	7.35 ± 1.36	9.81 ± 1.88	2.46	*P* < 0.001
PH3 (mm)	8.93 ± 1.16	11.06 ± 1.17	2.13	*P* < 0.001
AP3 (mm)	12.40 ± 1.24	14.34 ± 0.87	1.94	*P* < 0.001
AH4 (mm)	6.99 ± 1.32	9.24 ± 1.44	2.25	*P* < 0.001
PH4 (mm)	8.69 ± 1.25	10.99 ± 1.38	2.29	*P* < 0.001
AP4 (mm)	12.53 ± 1.35	14.39 ± 1.06	1.86	*P* < 0.001
ML (mm)	90.87 ± 7.59	104.79 ± 4.62	13.92	*P* < 0.001

### Measurement error

Our data had a normal distribution (*P* > 0.746), so paired sample *t* test could be applied. The results indicated that there were no significant differences between the mean of the measurements on different occasions *(P* > 0.05 for all six variables). The standard deviation ranged from 0.48 to 1.37 mm.

### Multiple regression analysis

In this study, MLI was chosen as a dependent variable and six factors were selected as independent variables. Statistical analysis data showed that two independent variables (AP3 and PH4) had a significant effect in the predicting of the dependent variable. AP3 and PH4 showed significant statistical correlation with MLI (*r* = −0.601 and −0.533, respectively, and *P* < 0.001 for both). The dependence of MLI on AP3 and PH4 can be expressed as follows:

MLI = 76.210-3.145(AP3) − 2.677(PH4).

In the present study, *R*^2^ was 0.577 and adjusted *R*^2^ was 0.549. In other words, the combination of AP3 and PH4 explained the variability of MLI by nearly 55%.

Using multiple regression menu in the PASS11 software (NCSS, LLC., Kaysville, Utah, USA) and by considering type one error of 0.05 and the sample size of 33, the power of this study was obtained as 0.98.

### Predictive accuracy

Table 2 lists the average errors between predicted and actual MLI and average errors of the absolute value in our model using the jackknife method and Mito et al.'s method (MM). The average error was calculated as *Σd/n*, where *d* is the difference between two registrations of a pair and *n* is the number of double registrations. According to this index, the value for our method was 0.149 mm, whereas the average error of MM was 5.87 mm. In addition, the two methods were compared using Dahlberg formula [(*Σd*^2^/2*n*)^1/2^]. The value of this error was 3.72 mm for our method and 7.53 for MM. Moreover, paired samples *t* test showed a statistically significant difference between our method and MM (*P* < 0.001 for both errors). So, the accuracy of our method indicated significant differences in comparison with MM.

**Table 2:** **Average errors ± SD between our model using jackknife method and Mito et al.'s method (MM)**[12]

	**Our method**	**MM**	***P *** **value**
Average error (mm)	0.149 ± 5.35	5.87 ± 9.02	*P* < 0.001
Average error (absolute value) (mm)	4.05 ± 3.41	8.4 ± 6.64	*P* < 0.001

## Discussion

Growth modification treatments require information on the growth potential. The orthodontist should be aware whether growth is happening and how much of the remaining incremental growth should be expected [13]. Moreover, evaluation of time of occurrence of this growth is also crucial [8].

As indicated by the results of several studies [3,4,7-12], the stages of cervical vertebral maturation are related to mandibular growth changes taking place during puberty. The main goal of the present study was to provide the orthodontist with an easy tool to determine the mandibular GP. This was to be accomplished by analyzing the changes of the cervical vertebrae on the lateral cephalometric radiograph of the patient head, a type of film used routinely in orthodontic diagnosis.

In this study, only girls were examined because of sex-dependent differences with regard to the timing of morphological changes in cervical vertebral bodies [6]. A group of 9- to 11-year-old girls was considered because usually at these ages, children refer to orthodontists. On the other hand, maturation indicators of brief duration are more informative than those of the longer duration [14]. A wide age range of the population may affect the correlation result because of the inability of skeletal maturity methods to detect changes in skeletal maturity precisely when the subjects are either too young or too old, i.e., too far ahead of or too far past the pubertal growth spurt [15]. Ar-Pog was used in this study as mandibular length because it is easily located; [16] the difference between the two stages was used as the MLI.

A stepwise regression analysis was used in this research to define prediction models that could be used to forecast individual future growth changes of the mandible. The stepwise method was used to select the explanatory variables. As the result of the statistical analysis on the present sample, two independent variables (AP3 and PH4) were significantly selected amongst variables studied in order to explain the dependent variable MLI by MLI = 76.21-3.145(AP3) − 2.677(PH4). AP3 and PH4 showed significant statistical correlation (*P* < 0.001) with MLI. The variability of the dependent variable, which is defined by *R*^2^, was 57.7% and adjusted *R*^2^ was 54.9%. According to the statistical rule, the number of samples must be at least twice as many as the number of independent variables [17]. The present study consisted of 33 cases which was a satisfactory number to make the regression coefficients, and *R*^2^ and adjusted *R*^2^ values truly represents the actual population.

Here, adjusted *R*^2^ is presented, which is a modification of *R*^2^ that adjusts for the number of explanatory terms in a model. Unlike *R*^2^, the adjusted *R*^2^ increases only if the new term improves the model more than what would be expected by chance. The higher *R*^2^ while there were fewer independent variables exhibits an easier method with better prediction accuracy [17].

Chen et al. [12], reported that the combination of AH3, AH4, and AP3 explained the variability of MLI (*R*^2^ = 61.35%). The reasons for the differences between their results and the present study can be racial factors in addition to the limited age range in our study. Furthermore, subjects in their study include both Class I and Class II subjects.

The longitudinal nature of this study allows determination of the prediction accuracy of our equation by best means. So, the actual growth can be determined individually in all subjects. As reported in several studies [2,4,7,8,10,11], the peak in mandibular growth occurs from CVMS II and CVMS III, and the duration of this peak interval in normal occlusion is 1 year [18]. In our study, all the subjects were in CVMS I or II at the time of first examination. The 24-month interval allowed reaching the CVMS III in nearly all of the cases. Thus, it can be said that the peak has occurred in the majority of subjects.

**Figure 2:**
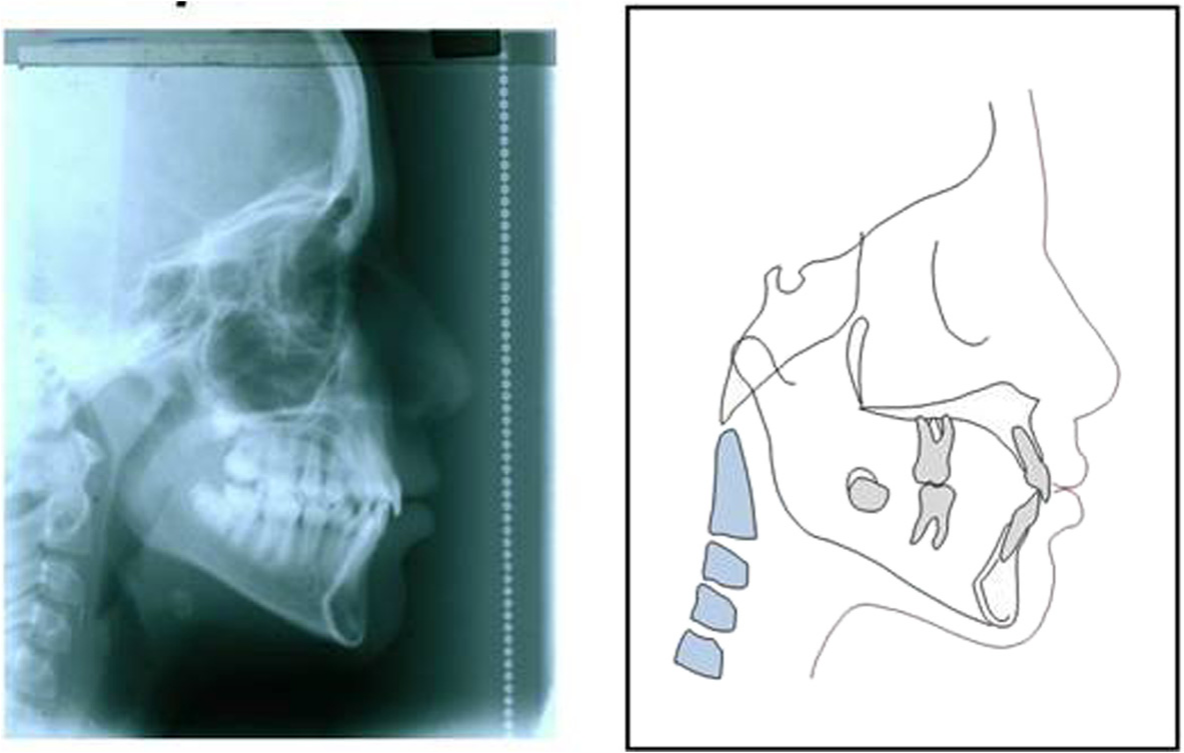
Pretreatment lateral cephalogram and tracing of the patient.

**Figure 3:**
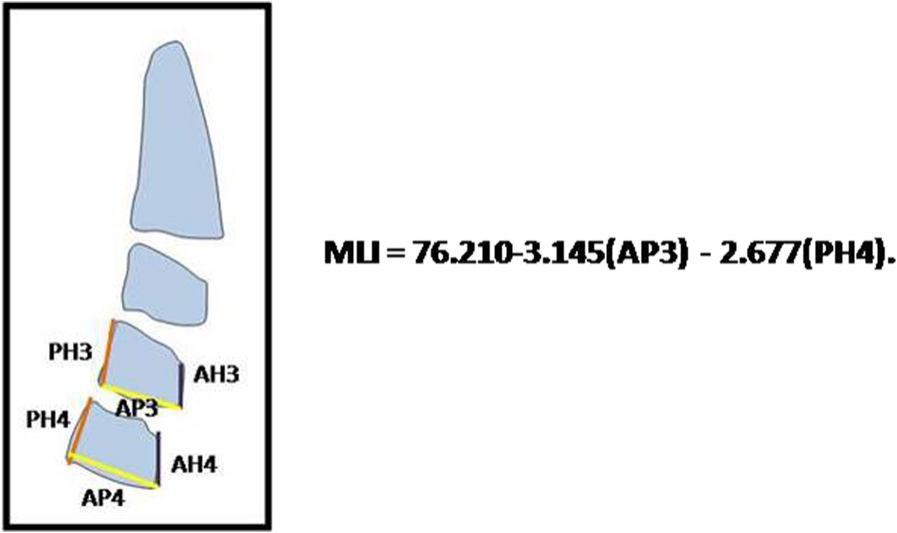
Cervical vertebral dimension and MLI of the 9-year-old patient.

**Figure 4:**
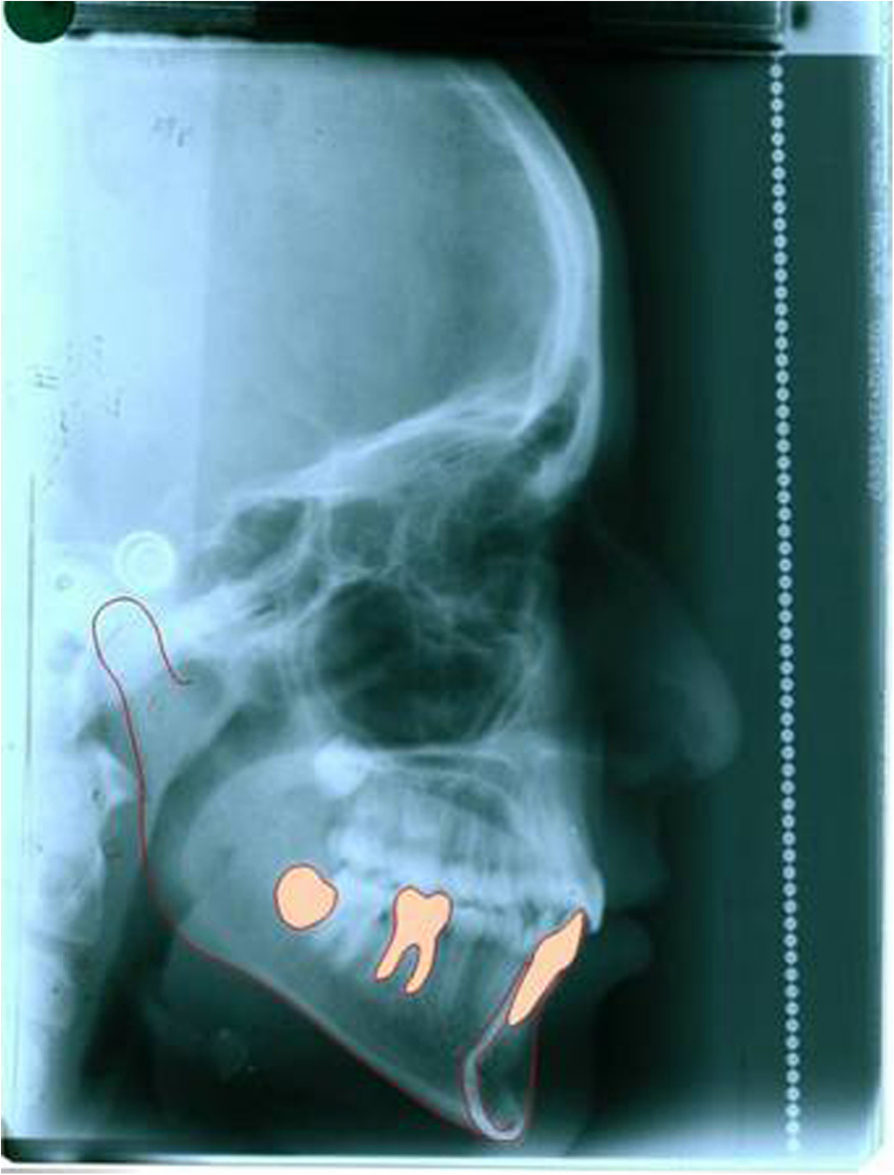
Post treatment lateral cephalogram and mandibular tracing of the patient.

**Figure 5:**
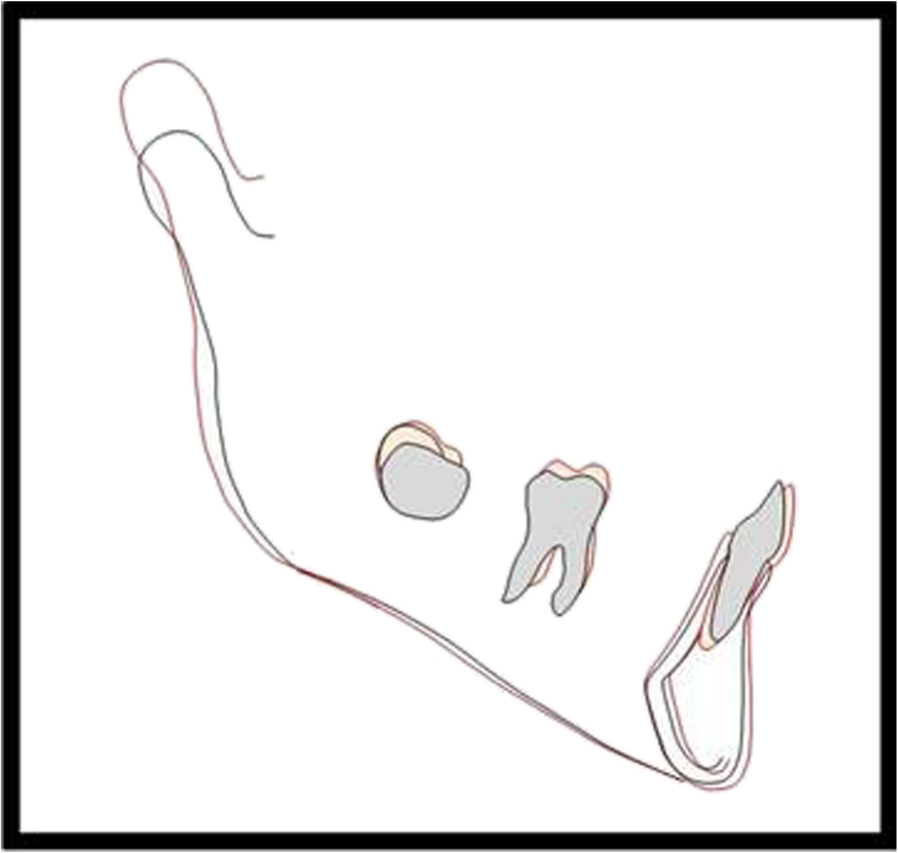
Superimposition of the pre and post treatment cephalograms of the patient. Note that the mandibular growth was in accordance with pretreatment prediction.

The formula obtained from this study can be easily adopted in the daily clinical practice as shown in Figures 2, 3, 4, and 5. S.J. is a 10-year and 2-month-old patient who was referred to our private practice in February 2008 with a chief complaint of her lower incisor crowding. Her parents were also concerned about her slightly prominent chin. Our formula can be useful in predicting further mandibular growth and discussing our prediction with the patient/parents. Lateral cephalogram was obtained, and mandibular growth increment was calculated. In March 2010, another cephalograms were obtained after finishing orthodontic treatment. After superimposition on the mandible, it turned out that our prediction was reasonably accurate.

We compared the predictive accuracy of our equation with the method of Mito et al. [11], which is suitable for predicting mandibular growth potential in skeletal Class I patients, and our method showed a better prediction accuracy. The reason for lower prediction accuracy for Mito et al.'s method in our subjects can be explained as follows:

1. The formula presented by Mito et al. [11] was designed for Japanese girls. Racial, environmental, and genetic differences between Japanese and Iranian girls can result in differences in developmental biologic clock.

2. The subjects consisted of a wider range in Mito et al.'s study [11] (7 to 21 years old) which included individuals before, during, and after growth spurt. However, in our study, the samples were in a more limited age range.

3. Our study included only subjects with Class I normal occlusion but Mito et al. [11] used Class II as well as Class I cases.

Our equation predicted the MLI in circumpubertal growth spurt, which is a golden period in regard to treatment efficiency [13].

In a study conducted by Fudalej and Bollen [19], the effectiveness of CVM method in 15 to 27 years old orthodontic patients in postpeak period was evaluated. They found this method as only modestly effective. This may seem controversial with the findings of our study; nevertheless, as they mentioned, there is some weakness in this mixed longitudinal study design, namely:

a) The unknown amount of late postadolescent growth

b) Although few but there were statistically significant differences between orthodontic patients and those with a normal occlusion (such as our samples) in terms of growth.

c) Data of both genders should not be combined when the aim is to present mean ages.

d) Furthermore, the samples analyzed by Fudalej and Bollen [19] were in a far later stage of CVM than ours.

### These may explain different conclusions

According to the longitudinal survey of Ball et al. [20], the most frequent stages at which mandibular increment occurs is CS4 which is compatible with stage II and III in the method by Baccetti et al. [7]. This is in complete agreement with our study in which most of the cases where in CVMS II through III. On the other hand, Ball et al. [20] concluded that it is not possible to predict peak maturation growth velocities by cervical vertebrae alone, this is a controversial issue. However, any controversies between two conclusions is due to differences in case samplings: our findings are based on 9 to 11 years old girls with Class I occlusion, whilst the study by Ball et al. [20] concentrated on 9 to 18 years old boys without any particular Class of Angle's classification.

In the current study, there are few limitations regarding age and gender of samples used as well as their ethnicity that is limited to Iranian girls. These limitations can be addressed in future studies.

## Conclusion

In this study, we established a new equation to predict mandibular growth and compared it with Mito et al.'s method [11]. The equation provided a useful method for predicting mandibular growth increment in Iranian girls, on the basis of only a single cephalometric radiograph.

## Competing interests

The authors declare that they have no competing interests.

## Authors’ contributions

MM and MN were responsible for acquisition of Data. AAB was responsible for the statistical analyses. HR and HR were responsible for conception and design of the study, interpretation of data and drafting the manuscript. All authors read and approved the final manuscript.
